# The Effect of Influenza Vaccination on COVID-19 Morbidity, Severity and Mortality: Systematic Review and Meta-Analysis

**DOI:** 10.21315/mjms2021.28.6.3

**Published:** 2021-12-22

**Authors:** Meysam ZEYNALI BUJANI, Mohammad BEHNAMPOUR, Nima RAHIMI, Tahereh SAFARI, Abdurrashid KHAZAEI FEIZABAD, Armaghan HOSSEIN SARBAZI, Marzieh BANIASADI, Nima REZAEI, Alireza ANSARI MOGHADDAM

**Affiliations:** 1 Student Research Committee, Zahedan University of Medical Sciences, Zahedan, Iran; 2 Interest Group of Coronavirus 2019 (IGCV-19), Universal Scientific Education and Research Network (USERN), Zahedan, Iran; 3 Department of Physiology, Zahedan University of Medical Sciences, Zahedan, Iran; 4 Department of Medical English, Zahedan University of Medical Sciences, Zahedan, Iran; 5 Department of Emergency Medicine, Zahedan University of Medical Sciences, Zahedan, Iran; 6 Research Center for Immunodeficiencies, Children’s Medical Center, Tehran University of Medical Sciences, Tehran, Iran; 7 Department of Immunology, School of Medicine, Tehran University of Medical Sciences, Tehran, Iran; 8 Network of Immunity in Infection, Malignancy and Autoimmunity (NIIMA), Universal Scientific Education and Research Network (USERN), Tehran, Iran; 9 Health Promotion Research Center, Zahedan University of Medical Sciences, Zahedan, Iran

**Keywords:** COVID-19, influenza vaccine, hospitalisation, mortality, ICU, prevention

## Abstract

Coronavirus disease 2019 (COVID-19) pandemic is rapidly developing worldwide with a high mortality rate. In this meta-analysis study, the effect of influenza vaccination on the prevention of COVID-19 and its consequences in patients were investigated. The systematic search for this study was performed from November 2019 to 25 November 2020, in the databases of Medline, PubMed, Scopus, Web of Science, Embase, Ebsco, Cochrane and medRxiv. Search terms used included COVID-19, coronavirus, SARS-CoV-2, covid, influenza, flu, grippe and vaccine. The present study examined the association between influenza vaccination and COVID-19 including COVID-19 infection, mortality, hospitalisation and intensive care unit (ICU) admission. Finally, the pooled estimates for different outcomes were calculated by the software for statistics and data science (STATA) version 15 and I^2^ was used to determine the heterogeneity. By analysing the data of articles, the pooled estimates of these data indicated that influenza vaccination could lower probability of COVID-19 infection up to 24% (OR = 0.77; 95% CI: 0.65, 0.91), of death up to 32% (OR = 0.68; 95% CI: 0.42, 1.11), of the hospitalisation up to 25% (OR = 0.75; 95% CI: 0.46; 1.23) and of admission to ICU up to 29% (OR = 0.71; 95% CI: 0.40, 1.27). Influenza vaccination can help decrease the COVID-19 infection and reduce hospitalisation and the need for ICU and mortality rates.

## Introduction

The newly found coronavirus has caused the coronavirus disease 2019 (COVID-19). In December 2019, the first case of the COVID-19 infection was reported in Wuhan, China (1). This virus was a new strain of coronavirus which rapidly spread all around the world (2). Hence, it was announced as a pandemic by the World Health Organization (WHO) in March 2020. Among about 69 million confirmed cases of COVID-19, over 1.5 million individuals have globally lost their lives due to the infection (3). It is estimated that 8 out of 10 patients manifest mild symptoms of COVID-19 and remaining two cases become severely critical (4). If this trend continues and no intervention is applied, it may affect the communities for several years and impose a heavy burden both on people and economy of countries, especially those with poor health systems (5).

Epidemiological data suggest that the elderly and people with underlying diseases such as obesity, diabetes, cardiovascular, respiratory and renal diseases are at a greater risk of infection (6). Moreover, the effect of this disease varies across different countries and ethnicities. The mortality rate depends on various factors including genetic, cultural, political and even social behaviours. Due to economic health policies, studying these issues requires extensive epidemiological investigations in different countries. Various studies indicate a wide range of symptoms, from asymptomatic infection to respiratory failure and death (4). Evidences reported in several articles suggest that social distancing, hand hygiene and mask protection could help disease prevention (7). By evaluating the effectiveness of the non-medical interventions of governments to reduce the prevalence of COVID-19, social distancing and traffic restrictions were recognised as the most important measures among non-medical interventions. Meanwhile, strategies such as drug treatment and vaccination are recently being investigated (7, 8).

Due to the onset of cold season and the simultaneous outbreak of influenza and COVID-19, the researchers have likened the period to a critical storm. The influenza epidemic usually begins in the cold seasons (9). One way to prevent this epidemic is to use the influenza vaccine. While, this vaccine is usually updated and re-prescribed every year, there is much unknown about how the cold season and the flu may affect COVID-19. Researchers wonder if the co-infection will have any effect on COVID-19 (10).

Studies showed that there is a possible relationship between the influenza vaccine and COVID-19. These two viral diseases are overlapping in different ways. Influenza vaccine was reported to help reduce the symptoms of COVID-19 and cause a less intense disease. Influenza vaccine has also been shown to induce the immune responses which respond to COVID-19 and help reduce its infection and subsequently decrease the symptoms (11, 12).

Due to the onset of cold season, the increasing prevalence of influenza and COVID-19, and the best of our knowledge, the absence of a systematic review or meta-analysis, the present study was designed to conduct a meta-analysis of the published findings. Public health decisions around the world should be made due to the best available evidence.

## Methods

The present study was designed due to the Preferred Reporting Items for Systematic Reviews and Meta-Analysis (PRISMA) guide (13).

### Search Strategy

The systematic search for this study was carried out in different databases including Medline, PubMed, Scopus, Web of Science, Embase, Ebsco, Cochrane and MedRxiv, both on peer review and non-peer review articles in all languages (with an abstract in English) from November 2019 to 25 November 2020. Medical subject heading (MeSH) terms and combined text were used to search in databases. Search keywords included COVID-19, coronavirus, SARS-CoV-2, 2019-nCoV, COVID, influenza, flu, grippe and vaccine.

### Data Collection and Synthesis of Data Extract

Firstly, two authors conducted a systematic search independently and after the articles were extracted, they were sent to the third author to check if any possible inconsistency occurred. Then, the full texts of selected articles were evaluated. The studies which reported the odds ratio (OR), relative risk (RR) and hazard ratio (HR) between the influenza vaccine and the COVID-19 were included. Moreover, the data were examined to ensure the ratios were not directly mentioned. References to these articles were also reviewed to ensure that relevant studies were not missed. While evaluating, special attention was paid to cohort, case-control, and cross-sectional studies, and studies with no valuable data, duplications, and qualitative reports of vaccine efficacy were excluded. The author’s name, year of publication, age and sex of subjects, and the outcomes of influenza vaccination and COVID-19 such as coronary heart disease, mortality, hospitalisation and intensive care unit (ICU) admission were examined.

Finally, random pooled effect OR for different outcomes was calculated by STATA version 15, and I^2^ was used to determine heterogeneity.

## Results

**Figure 1:**
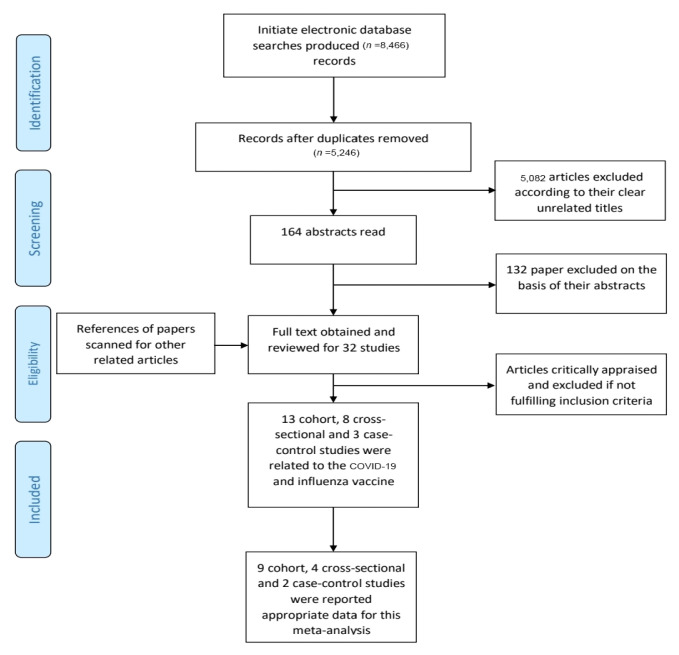
Flow diagram of the number of studies screened and included in the meta-analysis

After eliminating the duplicates, 5,246 papers were screened, of which 164 studies were found to have relative data to our investigation. By reviewing the full text of these 164 studies and considering the predetermined inclusion criteria, 15 articles [9 cohort studies (4, 14–21), four cross-sectional studies (22–25) and two case-control studies (26, 27)] were eventually selected, which had valuable and related data for meta-analysis (Figure 1). In general, more than 120,000 patients were investigated in our research. Table 1 presents the number of people, mean age, gender and the number of groups evaluated for influenza vaccination in each study.

**Figure 2:**
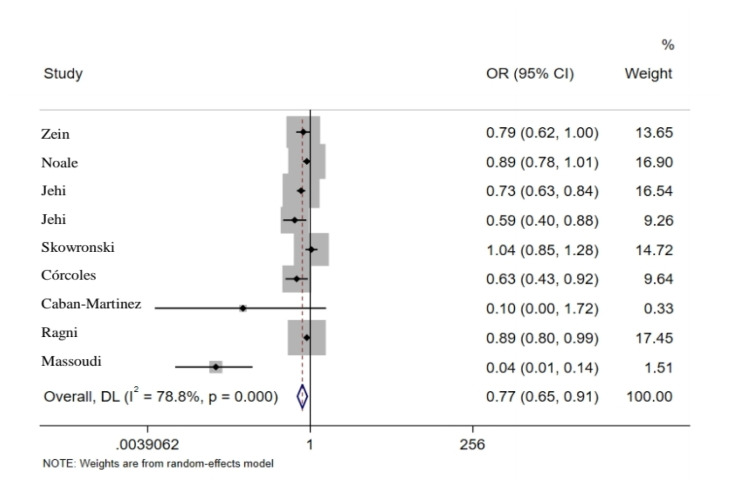
Association between influenza vaccination and COVID-19

All studies included in this meta-analysis were evaluated for the relationship between receiving the influenza vaccine and the probability of getting infected by COVID-19. Furthermore, the rate of mortality, hospitalisation and admission to ICU were analysed independently, if reported (Table 1). Random effect pooled estimates were used to assess the effectiveness of influenza vaccine on SARS-CoV-2. Eight studies with nine groups (one included two cohorts) reported new COVID-19 rates in people who had flu vaccination the previous year. The pooled OR of these data indicated that influenza vaccination reduced the rate of COVID-19 to 24% (OR = 0.77; 95% CI: 0.65, 0.91) (Figure 2) (14–17, 19, 25–27), where the pooled estimates of adjusted and non-adjusted studies showed a significant difference (*P* = 0.020) indicating 12% and 64% reduction, respectively. These eight articles were of different study types, including cross-sectional, cohort and case-control, which calculated pooled estimates in outcomes and indicated no significant difference.

**Figure 3:**
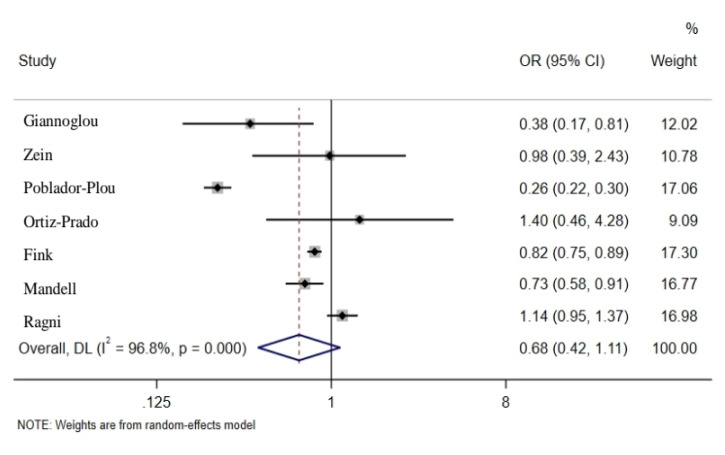
Association between influenza vaccination and mortality

**Figure 4:**
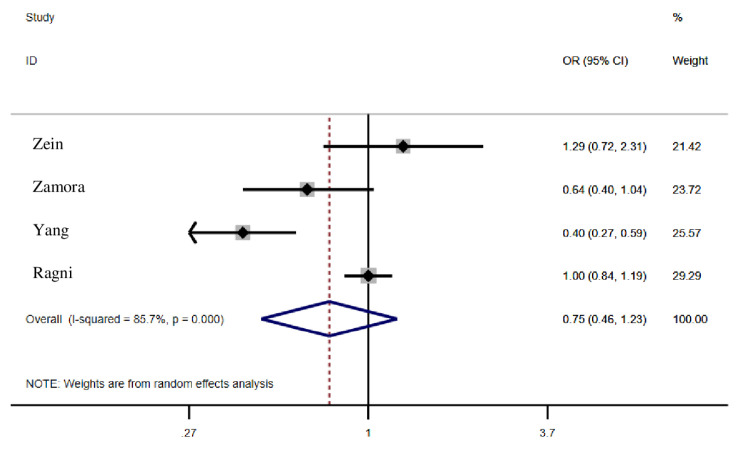
Association between influenza vaccination and hospitalisation

**Figure 5:**
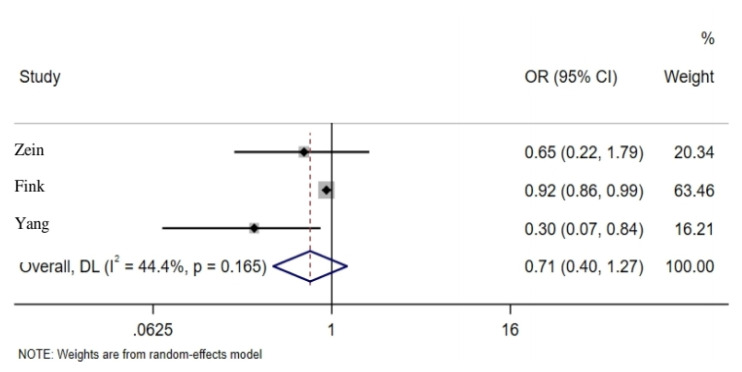
Association between influenza vaccination and ICU

Pooled estimates of seven studies which had reported the association between influenza vaccination and mortality rate in people with SARS-CoV-2 showed that vaccination could reduce the mortality rate in individuals up to 32% (OR = 0.68; 95% CI: 0.42, 1.11); however, it was not statistically significant (Figure 3). Moreover, the pooled estimates of studies which had reported adjusted and non-adjusted findings were not statistically significant (*P* = 0.17) (4, 14, 15, 18, 22–24). The articles were based on cross-sectional and cohort studies in which the pooled estimates in outcomes were calculated, indicating no significant difference. Four articles analysed the association between influenza vaccination and hospitalisation, where pooled estimates indicated a 25% (OR = 0.75; 95% CI: 0.46, 1.23) reduction among vaccinated individuals, which is not statistically significant (Figure 4). The pooled estimates of studies reporting adjusted and non-adjusted findings were not significantly different (*P* = 0.61) (14, 15, 20, 21). Three articles found a reduced ICU admission among subjects with COVID-19 who had received influenza vaccination where pooled estimates indicated a 29% reduction among vaccinated individuals; this rate was not, however, statistically significant [OR = 0.71; 95% CI: 0.40, 1.27). The difference among the pooled estimates of adjusted and non–adjusted groups were not significant (*P* = 0.10) (15, 18, 21) (Figure 5). Furthermore, one study reported a 20% reduction of mechanical ventilation for COVID-19 subjects who received influenza vaccination (18).

## Discussion

The present study results indicated that people with a history of influenza vaccination had up to a 24% reduction in COVID-19 infection. Furthermore, if COVID-19 infected the individuals, 25% hospital admission, 29% ICU admission and 32% mortality rate would decrease. Studies revealed a close relationship between influenza and COVID-19 (11); hence, the influenza vaccine might help body immunity and reduce the severity of COVID-19 (2).

The findings derived from the evaluated studies revealed that the influenza vaccine could have a protective role in recipients and could reduce the infection by 24%. Similarly, the protective effect of influenza vaccine on COVID-19 was confirmed so that the risk of infection would reduce from 12% to 64% (13–15, 17, 20, 24). However, one study (26) did not find an association between influenza vaccination and COVID-19 which is not in line with the findings of other studies. The difference can be due to the type of study which was a case-control. The control group in this study consisted of patients whose COVID-19 tests were negative but not negative for other respiratory diseases such as influenza and other viral diseases. This issue is effective in immunising people against COVID-19; hence, it could have affected the outcome of the study. Although there are no definite findings on the protective mechanism of influenza vaccine against COVID-19, some studies supported the hypothesis that vaccination against one microorganism may develop immune responses to other microorganisms (29).

Stimulation of the immune system by the vaccine and generation of trained innate immunity can justify the hypothesis. Moreover, since the SARS-CoV-2 and influenza viruses are similar in evolution, transmission and pathogenicity, the vaccine may induce inflammatory and antiviral reactions by establishing similar patterns in receptor identification (23). The remarkably high price of influenza vaccination in the past year caused it to be used more by the economically upper social classes, which, in turn, brings about healthier circumstances compared to the lower economic levels of society (30). Based on these findings, governments should consider strategies for the universal influenza vaccination because regarding the pandemic and increasing demand for the influenza vaccine, the demand may exceed production capacity (9).

The present study results indicated that influenza vaccination could reduce the mortality rate in COVID-19 patients by 32%; the rate, however, is not statistically significant. Studies have shown that this rate varies from 2% to 74% (4, 15, 18, 23, 24). However, in studies conducted by Ragni et al. (14) and Ortiz-Prado et al. (22), the findings showed that influenza vaccination had no effect on mortality rate in COVID-19 patients, while the findings of one study (14) confirmed that creating a subgroup based on the type of vaccine, the trivalent vaccine, could reduce the risk of death up to over 30%. Hence, the inconsistency can be explained by the type of vaccination which many studies have ignored. Moreover, the reason for the difference between one study (22) and other studies can be elucidated by the fact that the condition of about 99% of patients was not clear. According to figures released by the World Health Organization (WHO), more than 1,500,000 lives were lost from SARS-CoV-2 since the beginning of the new coronavirus pandemic to November, which is about 2.4% of all patients with COVID-19 (3). Due to the Center for Disease Control and Prevention (CDC), it was estimated that in the years 2018 and 2019, 490,600 people were hospitalised with influenza virus, with a death rate of 34,200 (100 cases per 100,000 people infected with influenza) (31). Comparing the mortality rate of the two diseases of corona and influenza, it was shown that the mortality rate in COVID-19 is approximately 2.3%, while in influenza, it is less than 1% (3, 31).

Numerous studies report a significant relationship between influenza and COVID-19 infection and increased mortality and morbidity, and increased health care demand; therefore, maximal influenza vaccination should be provided to reduce the burden of both diseases (as long as a definite vaccine is not yet available) and to prevent the risks of co-infection of these two diseases. Pooled estimates were analysed for eight studies which evaluated mortality rate in people who were examined for COVID-19 and influenza vaccination. It was found that influenza vaccination could reduce the probability of death in patients up to 37% and prevent the co-infection of the two diseases that have a simultaneous poor prognosis. The reduction in mortality rate could be due to the faster clearance of the SARS-CoV-2 virus, the prevention of virus diffusion to the lower lungs or the reduction of host’s destructive excessive inflammatory response (18). Excessive inflammatory response and cytokine storm in patients with COVID-19 play a critical role in increasing mortality (32). Besides, a study by Netea et al. (33) reported that inflammatory cytokines produced in the body before the acute onset of disease could be beneficial in the mild phase of disease. Consequently, vaccination by creating inflammatory cytokines before the acute onset of COVID-19 disease might boost lowering the severity of the disease.

Due to a study in the United States, the COVID-19 hospitalisation was reported 164.5 per 100,000 people (34). Studies indicate that the influenza vaccine is effective in the rate of hospitalisation of people with COVID-19. Performing statistical calculations related to four studies suggested that hospitalisation of influenza vaccinated individuals could decrease by up to 25%; however, this finding was not statistically significant. In different studies, this rate varies from 36% to 60% (20, 21), which suggests that influenza vaccination could prevent the acute phase of COVID-19 and decrease hospitalisation; hence, it reduces the burden of disease on individuals. Moreover, vaccination reduces influenza hospitalisation as well as costs required for the health demand and the need for hospital beds, which are big problems in different countries. However, two studies by Ragni et al. (14) and Zein et al. (15) indicated that there is no significant relationship between vaccination and hospitalisation in COVID-19 patients. Although no correlation was reported in the study (21), the results showed that by creating subgroups due to vaccination time (October to November 2019 and December 2019 to March 2020), the late vaccination group reduced hospitalisation by about 30%. As a result, the duration after vaccination could affect the outcome of disease. The study by Fink et al. (18) is also confirmatory, but it should be noted that even if the positive effects of vaccine were confirmed, this immunity could not remain for a long time (35). In another study (15), the vaccination duration of patients was not mentioned, which could be a reason for the difference between these findings and other studies.

In various studies, the rate of ICU admission for COVID-19 patients was reported from 5% to 15.4%, and the need for a ventilator is from 2.3% to 14.9% (36, 37). It was shown that the influenza vaccine could reduce the need for ICU hospitalisation by 29%. In other studies, this rate varies from 8% to 70% for ICU admission (15, 18, 21). Considering the COVID-19 pandemic, the growing need for the patients to be admitted to the ICU and connected to a ventilator, and the lack of ICU beds and ventilators, a general vaccination strategy can be recommended to governments to help reduce patients’ demands and increase capacity for more critical patients.

## Limitations

One of the limitations of this study was that there was a lot of heterogeneity among the sample sizes of different studies (from 203 to 198,828 patients). In many studies, patients were not monitored long enough and patient information was recorded at specified intervals. Furthermore, many studies have evaluated vaccination as a side component, so it was not possible to determine subgroups based on similar studies in similar conditions. Furthermore, several studies did not specify the time intervals and type of influenza vaccine that could affect study results. Another limitation is the lack of clinical trial studies that could be performed to more accurately assess the effectiveness of the influenza vaccine on the COVID-19 or by designing case-control studies with a more accurate methodology than the current studies. There are other systematic studies in this field, but there is no study that meta-analyses these consequences.

## Conclusion

Because the influenza crisis coincides with the COVID-19 pandemic, the influenza vaccine can help reduce the incidence of COVID-19 disease and reduce the hospitalisation rate, the need for ICU and mortality rate. It is also suggested that governments and politicians contribute to the current situation and increase the capacity of ICU beds for patients with more critical conditions by considering strategies for universal vaccination of individuals, along with other preventive measures.

## Figures and Tables

**Table 1:** Main characteristics of included studies in the meta-analysis

First author and year	Type of study	*N*	Influenza vaccine *n* (%)	Sex		Age	Setting	Infection		Hospitalisation		Mortality		ICU		MV	
	
				M *n* (%)	F *n* (%)			OR	95% CI	OR	95% CI	OR	95% CI	OR	95% CI	OR	95% CI
Zein, 2020 (15)	Cohort	18,868	4,138 (31.3%)	5,701 (43.1%)	7,519 (56.9%)	Median	Laboratory-verified COVID-19	0.79^1^	0.62–1	1.29^1^	0.72–2.31	0.98^1^	0.39–2.43	0.65^1^	0.22–1.79	-	-
Noale, 2020 (16)	Cohort	198,828	2,246 (33.6%)	80,167 (40.3%)	118,661 (59.7%)	48 (14.7)	Participants were recruited via social media	1.02	0.91–1.15	-	-	-	-	-	-	-	-

								0.89^2^	0.78–1.01								
Jehi, 2020 (17)	Cohort	11,672	6,324 (54.2%)	4,607 (39.5%)	7,065 (60.5%)	-	Laboratory-verified COVID-19	0.73	0.63–0.84	-	-	-	-	-	-	-	-
	Cohort	2,295	358 (15.6%)	969 (42.2%)	1,326 (57.8%)	-	Laboratory-verified COVID-19	0.58	0.39–0.87	-	-	-	-	-	-	-	-
Poblador-Plou, 2020 (28)	Cohort	4,412	1,732 (39.3%)	1,819 (41.2%)	2,593 (58.8%)	67.7 (20.7)	Laboratory-verified COVID-19	-	-	-	-	0.25	0.21–0.30	-	-	-	-
Fink, 2020 (18)	Cohort	92,664	28,819 (31.1%)	53,005 (57.2%)	39,659 (42.8%)	-	Clinically and molecularly confirmed COVID -19	-	-	-	-	0.82	0.75–0.89	0.92	0.86–0.99	0.80	0.74–0.87
Vila-Córcoles, 2020 (19)	Cohort	79,071	704 (45.5%)	37,620 (47.6%)	41,451 (52.4%)	65 (11.3)	Laboratory-verified COVID-19	HR 0.63^3^	0.43–0.92	-	-	-	-	-	-	-	-
Murillo-Zamora, 2020 (20)	Cohort	740	118 (15.9%)	424 (57.3%)	316 (42.7%)	43.7 (14.9)	Laboratory-verified COVID-19	-	-	0.64	0.40–1.04	-	-	-	-	-	-
Yang, 2020 (21)	Cohort	2,005	214 (10.7%)	798 (39.8%)	1,207 (60.2%)	43.6 (17.7)	Laboratory-verified COVID-19	-	-	0.35	0.24–0.49	-	-	0.17	0.04–0.47	-	-
	
										0.4^4^	0.27–0.59			0.3^4^	0.07–0.84		
Ragni, 2020 (14)	Cohort	17,608	5,427 (30.8)	7,889 (44.8%)	9,710 (55.2%)	-	Laboratory-verified COVID-19	1.26	1.17–1.34	HR 1.78	1.53–2.07	HR 3.81	3.21–4.51	-	-	-	-

								0.89^5^	0.80–0.99	1^6^	0.84–1.19	1.14^6^	0.95–1.37				
Skowronski, 2020 (26)	Case control	6,410	1,943 (30.3%)	-	-	-	Laboratory-verified COVID-19	1.17	0.97–1.40	-	-	-	-	-	-	-	-

								1.04^7^	0.85–1.28								
Massoudi, 2020 (27)	Case control	261	90 (34.5%)	141 (54.1%)	120 (45.9%)	39.52	Clinically verified COVID-19	0.04	0.01–0.14	-	-	-	-	-	-	-	-
Ortiz-Prado, 2020 (22)	Cross-sectional	9,468	84 (0.9%)	5,247 (54.4%)	4,221 (44.6%)	-	Laboratory-verified COVID-19	-	-	-	-	RR 1.40	0.46–4.28	-	-	-	-
Giannoglou, 2020 (23)	Cross-sectional	512	130 (25.4%)	317 (61.9%)	195 (38.1%)	60.4 (18.2)	-	-	-	-	-	1.59	0.95–2.66	-	-	-	-

												0.38^8^	0.17–0.81				
Mandell, 2020 (24)	Cross-sectional	90,000 <	recorded vaccination status data for more than 36,000 (about 40%)	-	-	-	-	-	-	-	-	0.73	0.58–0.91	-	-	-	-
Caban-Martinez, 2020 (25)	Cross-sectional	203	35 (18.9%)	188 (93.5%)	13 (6.5%)	Median	Laboratory-verified COVID-19	0.10	0.005–1.71	-	-	-	-	-	-	-	-

Notes:

1 = adjusted;

2 = adjusted for sex, age, education, Italian area of residence, dichotomised self-reported diseases, smoking status and contact with confirmed COVID-19 cases;

3 = adjusted for sex, age, history of vaccinations and comorbidities;

4 = adjusted for race, age, gender, hypertension, diabetes, COPD, obesity, coronary artery disease and congestive heart failure;

5 = odds ratio adjusted for age, comorbidity, and time of execution of the swab test over the pandemic period;

6 = adjusted for age, sex, comorbidity;

7 = adjusted for age group, province, specimen collection interval, calendar time and season;

8 = adjusted
